# Impact of interprofessional education for medical and nursing students on the nutritional management of in-patients

**DOI:** 10.3205/zma001219

**Published:** 2019-03-15

**Authors:** Benedikt Braun, Matthias Grünewald, Renate Adam-Paffrath, Bärbel Wesselborg, Stefan Wilm, Lena Schendel, Matthias Hoenen, Karsten Müssig, Thomas Rotthoff

**Affiliations:** 1 Heinrich-Heine-University Düsseldorf, Medical Faculty, Düsseldorf, Germany; 2 University Hospital Düsseldorf, Bildungszentrum, Düsseldorf, Germany; 3 Fliedner Fachhochschule Düsseldorf, University of Applied Sciences, Düsseldorf, Germany; 4 Heinrich-Heine-University Düsseldorf, Institute for General Practice, Düsseldorf, Germany; 5 Heinrich-Heine-University Düsseldorf, Medical Faculty, Division of Endocrinology and Diabetology, Institute for Clinical Diabetology, Düsseldorf, Germany; 6 German Diabetes Center, Leibniz Center for Diabetes Research at Heinrich Heine University Düsseldorf, Düsseldorf, Germany; 7 German Center for Diabetes Research (DZD), München-Neuherberg, Germany; 8 University Augsburg, Medical Faculty, Department for Medical Education and Educational Research, Augsburg, Germany

**Keywords:** patient safety, interprofessional collaboration, interprofessional education, malnutrition, nutrition management, quality improvement, inquiry-based learning

## Abstract

**Introduction: **Despite its frequency, malnutrition is underestimated in its importance for morbidity and mortality. Interprofessional nutrition management can improve patient safety and clinical outcomes. An interprofessional education is considered as the basis for good team cooperation. So far, little data is available on the effects of interprofessional education on measurable outcomes for patients. The objective is to determine to what extent student feedback leads to a change of in-patient nutritional management for a selected internal medical ward.

**Methodology:** In a teaching project based on the method of research oriented learning, medical and nursing students conducted an analysis of the nutritional situation of patients and developed individual treatment plans. The students orally reported their findings to the care teams as well as via a poster presentation to decision-makers of the clinic. A prospective cohort intervention study was conducted to assess the nutritional status of patients before and after student interventions using established screening tools. Differences were tested using t-test and Fisher’s exact test. Institutional consequences for nutrition management were recorded descriptively. The teaching unit was evaluated by the students before and after.

**Results: **Malnutrition was found in 59% of patients. Inspired by student feedback, institutional consequences followed: a) routine inpatient screening using Nutritional Risk Screening; and b) the use of pie charts to estimate food intake.

**Conclusion:** The feedback from the results of student interprofessional cooperation led to a sensitization of decision-makers and enabled new measures to improve nutritional management. These can increase patient safety.

## Zusammenfassung

**Einleitung: **Mangelernährung wird trotz ihrer Häufigkeit in ihrer Bedeutung für Morbidität und Mortalität unterschätzt. Ein interprofessionelles Ernährungsmanagement kann die Patientensicherheit und das klinische Outcome verbessern. Als Grundlage für eine funktionierende Zusammenarbeit im Team wird eine interprofessionelle Ausbildung angesehen. Bisher liegen kaum Daten bezüglich der Effekte von interprofessioneller Ausbildung auf messbare Ergebnisse für Patienten vor. Für eine ausgewählte internistische Station sollte untersucht werden, inwieweit eine studentische Rückmeldung zu einer Veränderung des stationären Ernährungsmanagements führt.

**Methodik: **Medizin- und Pflegestudierende führten in einem Lehrprojekt, basierend auf der Methode des Forschenden Lernens, bei Patienten eine Analyse der Ernährungssituation durch und entwickelten individuelle Therapiepläne. Die Studierenden meldeten ihre Ergebnisse in einem Gespräch an die Versorgungsteams sowie in einer Posterpräsentation an Entscheidungsträger der Klinik zurück. Es wurde eine prospektive Kohorten-Interventionsstudie mit Erfassung des Ernährungszustandes von Patienten vor und nach den studentischen Interventionen mit etablierten Screeninginstrumenten durchgeführt. Unterschiede wurden mittels t-Test und exaktem Fisher-Test getestet. Institutionelle Konsequenzen für das Ernährungsmanagement wurden deskriptiv erfasst. Die Lehreinheit wurde von den Studierenden vorher und nachher evaluiert.

**Ergebnisse: **Bei 59% der Patienten wurde eine Mangelernährung festgestellt. Angeregt durch die studentischen Rückmeldungen folgten als institutionelle Konsequenzen: a) ein routinemäßiges stationäres Screening mit dem Nutritional Risk Screening und b) die Verwendung von Tellerdiagrammen zur Abschätzung der Nahrungszufuhr.

**Schlussfolgerung: **Die Rückmeldung der Ergebnisse aus der studentischen interprofessionellen Zusammenarbeit führte zu einer Sensibilisierung von Entscheidungsträgern und ermöglichte neue Maßnahmen zur Verbesserung des Ernährungsmanagements. Diese können die Patientensicherheit steigern.

## 1. Introduction

### 1.1. Interprofessionality and Patient Safety

Poor communication is a major cause of losses in the health care system [1]. Many errors along with ineffective use of resources are based on a lack of cooperation, poor coordination [2] and communication, e.g. in the medication of patients [3]. These benefit demonstrably from improved interprofessional cooperation and coordination of involved staff in the health care system [4], [5], [6], [7], [8], [9]. Thus interprofessional collaboration (IPC) can result in improved clinical outcomes and increased patient safety [8], [9], [10].

IPC in health care is a process in which different occupational groups work together to achieve common goals, such as safe, efficient and effective care [11], [12], [13]. Interprofessional Education (IPE) is regarded as an important basis for good team cooperation [14], [15], [16]. Early interprofessional experiences during training lead to a greater willingness to become an active member of an interprofessional team later on in the workplace [17]. By engaging with the self-understanding of other professions, mutual appreciation can be achieved through a joint learning process. The socialization process of the students, the perception of their own role in an interprofessional working environment, can thus be promoted [18]. The basis for this is the Allport’s Contact Hypothesis [14], [19], [20], [21] according to which stereotypical opinions about other professions can be overcome by early contact, for example as part of joint training [18]. 

Subjects with clinical relevance for patient safety, with direct practical relevance and large overlaps for the participating professions are suitable for interprofessional learning and work [22]. Accordingly the topic of malnutrition was chosen for the present study. So far, little data is available on the effects of interprofessional education on measurable outcomes for patients [23], [24].

#### 1.2. Malnutrition 

Despite its frequency, malnutrition is underestimated in its importance for morbidity and mortality [25]. Amongst the elderly in hospitals or nursing homes, the prevalence is 15-60% [25], [26]. Other surveys show a prevalence of 10-50% in hospitalized patients regardless of age [27], [28], [29]. Malnutrition occurs with inadequate food intake, increased demand, limited absorption, and altered nutrient use [26]. It constitutes a subacute or chronic condition that predisposes patients to disease, hampers recovery, and worsens clinical outcomes in terms of morbidity and mortality [28], [30], [31], [32]. Malnourished patients have longer hospital stays and an increased need for care and associated higher costs [28], [33]. Despite the importance of nutritional status in hospitalized patients, establishing the nutritional status is often neglected [28]. In order to ensure patient safety, the emphasis should be shifted from the treatment of a disease to the prevention of its manifestation [9]. This also applies to in-patient nutrition management. Interprofessional nutrition management can improve patient safety and clinical outcomes [9] by sharing tasks and responsibility [2].

#### 1.3. Hypothesis

The aim of the present study was to investigate to what extent the student work results from an interprofessional teaching project (using the method of research-oriented learning) at a selected internal medicine ward of the University Hospital Düsseldorf (UKD) lead to a change in in-patient nutrition management. The hypothesis was that student feedback leads to an improved detection and documentation of malnutrition and thus serves as a basis for increased patient safety. 

## 2. Methodology

**Figure 1:**
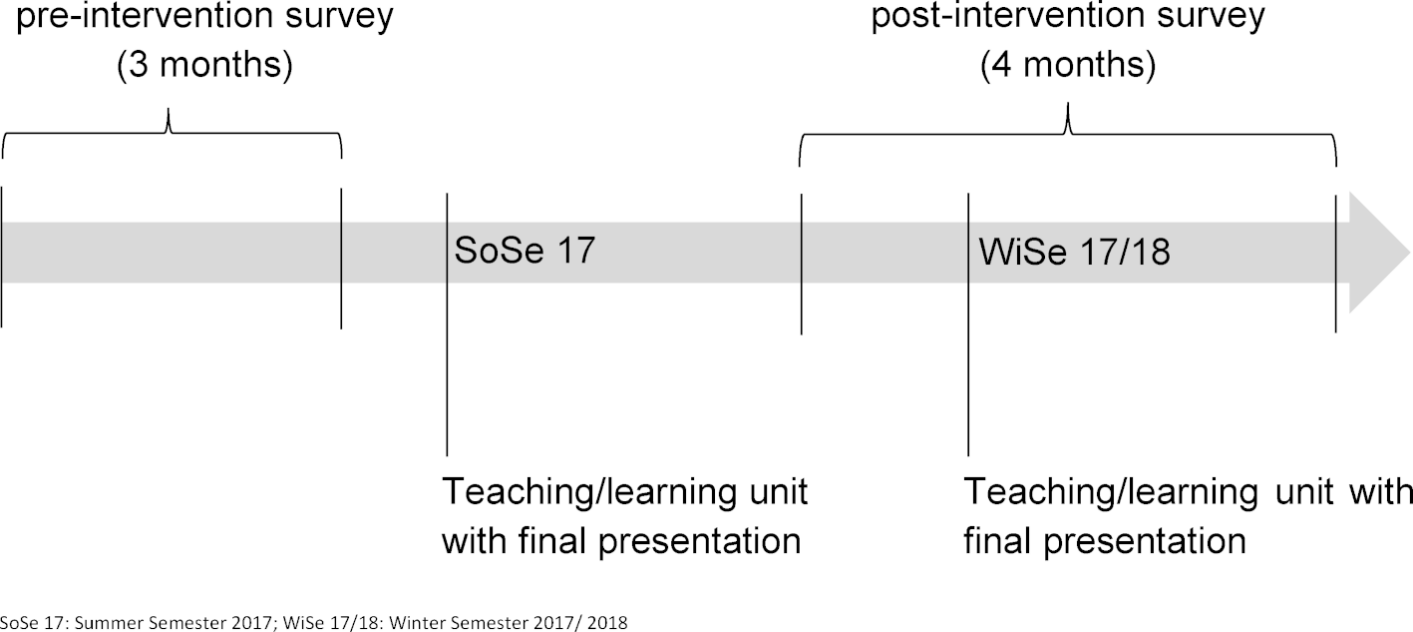
Time line of the teaching unit and the survey periods

In order to demonstrate the effects of the unit on in-patient nutrition management, a prospective, hypothesis-testing cohort intervention study was conducted at an internal medicine ward of the UKD. It consisted of a three month pre-interventional and a four month post-interventional analysis of the nutritional status of patients and an analysis of the current state of interprofessional nutrition management. The time line is shown in figure 1 (Fig. 1).

### 2.1. Interprofessional Teaching and Learning Unit

#### Concept of the Teaching and Learning Unit

In 2016 teachers and students of medicine, nursing and health sciences jointly developed the course “Interprofessional nutrition management in inpatient and outpatient care – *Stop Malnutrition!*”. The teaching and learning unit lasts 30 semester hours and is a compulsory optional subject in the medical curriculum of the Medical Faculty of the Heinrich Heine University of Düsseldorf (HHU) and part of the the compulsory curriculum in the bachelor’s degree program in Nursing and Health at the Fliedner Polytechnic. The project was funded by the Robert Bosch Foundation and carried out over two semesters. 

The nutrition education initiatives described in the literature are rarely directly related to patients [34]. In the project described here, the university didactic method of research-oriented learning was applied to the actual field of care [35]. The implementation in the field requires an intensive, mutual exchange and seemed to be the best way for students to learn with each other, from each other and about each other [17]. The learning process is based on a research cycle (hypothesis – research design – implementation – evaluation – mediation – application) [35] (see table 1 (Tab. 1)).

##### Implementation of the Teaching and Learning Unit

In the summer semester 2017, *n*=18 nursing students and *n*=12 medical students participated in the course, in the winter semester 2017/18, *n*=13 nursing students and *n*=10 medical students. In the summer semester, an interprofessional group of 3 students was assigned to the selected internal medicine ward of the UKD, and in the winter semester two groups of 3 students each. The remaining students were assigned to an academic teaching hospital as well as in the out-patient sector, and not included in the present study.

After preparatory key note lectures and group work phases on the subject of nutrition, taking into account the different domain perspectives, the students examined the nutritional management of real patients in small interprofessional groups of 2-4 participants at an internal medicine ward of the UKD. Following an analysis of individual nutritional status and nutritional conditions of each patient using validated questionnaires, students were asked to develop individualized treatment plans. 

At the end of the practical phase, the students reported their work results in a personal conversation with the professional teams of the ward. At the end of the teaching project, a final event was held each semester in which the students presented their research results, treatment plans, suggestions for optimizing nutrition management and reflections on the importance of interprofessional cooperation in a poster presentation. Decision makers of the involved institutions were also invited to the final presentation. 

##### Evaluation of the Teaching and Learning Unit

Before and after the teaching and learning unit, the students conducted a voluntary evaluation. On a Likert scale of 1 (I totally agree) to 5 (I totally disagree), participants were asked to rate the importance and expectations of the effects of interprofessional collaboration. The evaluation questions were taken from the German-language version of the *University of the West of England’s Interprofessional Questionnaire* (UWE-IP) [36]. 

#### 2.2. Situation analysis of in-patient nutrition management

##### Screening for malnutrition with validated questionnaires

In order to record possible changes in nutritional management after the teaching unit was carried out, an analysis of the current state of nutritional management was first carried out at the internal medicine ward. To assess malnutrition, patients were assessed using two published and validated questionnaires. The *Mini Nutritional Assessment* (MNA) [37] and the *Grazer Malnutrition Screening* (GMS) were used [38], [39]. The survey was carried out exclusively by a person instructed for this purpose (doctoral student in human medicine). In order to avoid study-related behavioral changes of the personnel in the context of the survey, ward doctors and nursing staff was blinded during pre- and post-analysis. The ward in question was relocated to a different building between the two analysis phases, which was accompanied by a reduction in the number of beds (18 beds pre-, 14 beds post-intervention). The post-intervention survey was therefore conducted over four months in order to investigate comparably large cohorts. 

##### Document analysis

In the event of a positive malnutrition test in at least one of the two questionnaires, the physician’s patient file and the patient’s medical records documenting nutritional management of both professions were analyzed by the doctoral student using a newly developed guideline (see attachment 1 ). For the preparation of the guideline, nutritionally relevant items were taken from the patient’s medical records, the doctor’s instructions, the ward round protocol, the nursing documentation sheet, the Complex Care Measures Score (PKMS), the form for taking patient history, and the nursing commentary sheet. The guideline was discussed and validated by an interprofessional team of nursing, health science, nursing education and physicians (diabetology and general practice). Before being used in practice, the guideline was independently applied to the patient records/files of three patients by two persons in the clinical setting. According to Altman [40], [41], there was a very high agreement in document analysis, Cohen's Κ=0.9, 95% confidence interval [0.826; 0.966]. The prerequisite for inclusion of the patients in the survey was a minimum existing in-patient stay of three days. Participation in the screening was voluntary for the patients. The consent was verbally requested by the investigator prior to the survey. However, there were problems with the use of the questionnaires in patients who were not lucid due to advanced dementia [25]. However, dementia is an important risk factor for malnutrition [31], [42]. In these cases, screening with the MNA was performed according to the authors’ recommendations based on their own impressions [43]. For the GMS there are no recommendations for use in dementia on the part of the authors. Therefore, screening was omitted in this case. 

#### 2.3. Survey of the effects of the teaching and learning unit on nutrition management

After completing the teaching unit, patients were again screened for malnutrition with the above mentioned questionnaires and through document analysis. The survey of institutional consequences was carried out by questioning the teachers, hospital management, the head of the central kitchen of the UKD, the head of the dietary kitchen and the ward physicians and nurses of the ward in question.

#### 2.4. Statistics

Statistics were calculated using IBM SPSS Statistics 24. For the comparison of the sum values an independent, two-sided t-test was carried out. To compare the frequency with which individual items were reported within the two survey periods, an exact Fisher test was used instead of a chi-square test because the expected cell frequency was <5 in many cases. For the rejection of the null hypotheses, a significance level of p=0.05 was chosen.

## 3. Results

### 3.1. Determined prevalence of malnutrition

The prevalence of malnutrition was comparable in the pre- and post-intervention cohorts (see table 2 (Tab. 2)). To illustrate the comparability of the two cohorts, demographic data such as age and sex of the patients were collected. They are listed in attachment 2 .

#### 3.2. Quantitative measurement results

Using the guide, the frequency of entries and information regarding nutrition management was collected in the interprofessional documentation of doctors and nursing staff. In the post-intervention cohort, significantly more information was found on nutritional management (*M*=9.3, *SD*=2.9, *SE*=0.3) than in the pre-intervention cohort (*M*=7.9, *SD*=3.1, *SE*=0.3; *t*(196)=-3.15, *p*=0,002, two-sided test). 

A change in frequency was found in the data on “Thirst assessment” (*p*<0.001) and “Appetite assessment” (*p*<0.001). In contrast, 85% of the variables collected through using the interprofessional documentation guide on nutrition management did not change. 

#### 3.3. Institutional consequences of feedback from the first course (SoSe 2017)

At the time of the pre-intervention analysis, there was no routine screening of the nutritional status at the relevant internal medicine ward of the UKD. Through the summer semester student feedback, the attention of hospital management was raised regarding the topic of nutrition management and subsequently routine screening of the nutritional status of patients was introduced. It uses *Nutritional Risk Screening* (NRS 2002), which is recommended in the guidelines of the European Society of Clinical Nutrition and Metabolism (ESPEN) [44]. 

#### 3.4. Institutional consequences of feedback from the second course (WiSe 2017/18)

There were also changes in the catering management supply structure. The distribution of meals and the clearing of the dishes is carried out on the wards of the UKD by service personnel. The students found that during an in-patient stay, it was not possible to get precise information on the eating habits of patients and therefore in the final presentation of the winter semester suggested the use pie charts. This would allow service personnel to record the amount of daily food intake of patients for each meal and to pass those assessments on to doctors and nursing staff. If necessary, supportive nutritional therapy could then be initiated immediately. On the basis of this student feedback, the clinic management called a meeting with the employees of diet counseling, the diet kitchen, kitchen management of the UKD, the head of service personnel as well as the nursing staff and doctors of the ward. The pie chart was implemented in February 2018. A modified form of published pie charts [45] was used for this purpose (see attachment 3 ).

#### 3.5. Evaluation results of the students 

From the evaluation of the students of both courses it can be seen that the students rate the interprofessional education and the consequences for the later professional cooperation positively (see table 3 (Tab. 3)). 

## 4. Discussion

### 4.1. Quality improvement through inquiry-based learning

The introduction of screening measures and the use of pie charts provided the impetus for direct quality improvement in the nutritional management of patients with malnutrition. This laid the foundation for improving patient safety. This is particularly important in the light of the fact that this impetus emanated mainly from the students as part of the teaching unit and that quality-oriented education and training units in the field of practice are said to play a key role in increasing patient safety [46]. 

The concept presented here goes beyond those previously presented, especially theoretically conceived teaching projects on quality improvement and patient safety [47]. By applying the method of research-oriented learning according to Wildt [35], the students had the opportunity to directly acquire and test interprofessional skills in the field of practice by analyzing patient cases from different specialist perspectives and developing an interprofessionally coordinated treatment concept. 

#### 4.2. Clinical relevance of the selected topic of the teaching and learning unit 

The results of this study also highlight the relevance of malnutrition to quality of care and patient safety. The surveyed proportion of 59% of patients screened as malnourished is in line with the results of other studies indicating prevalence rates of 30-60% [25], [27], [28], [39]. Overall, it is clear that a significant proportion of hospitalized patients are at risk of malnutrition and at risk from the associated consequences [28], [30], [31], [32], [33]. Our data highlights the recommendation [27], [48] of routine screening for malnutrition within 48 hours of admittance to a ward. Future teaching units should therefore focus more on care-related issues and patient safety issues. 

#### 4.3. Sensitization of hospital management to institutional consequences 

The development and implementation of the teaching project has made the hospital’s management more aware of the issue of malnutrition and led to an interprofessional discussion on the quality of nutritional management and patient safety. In addition to the hospital management, senior physicians and ward doctors, the nursing staff of the ward concerned, nutritional counseling employees, the dietary kitchen, the management of the service staff and kitchen management of the UKD were also involved. The collegial atmosphere in which all parties worked together on equal terms was highly valued and emphasized positively by all the professional groups involved. This is seen as fundamental to the establishment of new interprofessional relations [13] and may lead to an improvement in interprofessional cooperation [12]. 

#### 4.4. Factors influencing the quantitative results 

However although there were increases in the medical documentation of the items thirst and appetite, it cannot be assumed that these are directly attributable to the student feedback. It cannot be ruled out that the observed differences are due to the different documentation behaviors of the ward’s physicians.

The fact that no significant change could be detected in the other variables raised by the guideline (e.g. BMI, food and diet form) may be due to higher staffing variability in care as a result of the shift system. This can make communication more difficult between the professional groups involved [16]. Inadequate communication, lack of conscientiousness [12] and lack of time [39] make comprehensive interprofessional cooperation difficult.

In principle, it can be assumed that both the medical and nursing staff had little experience and knowledge of nutrition, malnutrition and nutrition management [28], [49]. In addition, a one-time feedback per semester by students is very likely insufficient. Repeated interprofessional reflection, e.g. during a ward meeting could lead to increased implementation, clarification of responsibilities amongst the various professional domains and thus to more effective interprofessional cooperation [50]. Following additional staff training on nutrition, greater effects on the quality of care could be expected [31]. Nutritional management which at that point would be interprofessional would then result in increased patient safety [9].

#### 4.5. Limitations and weaknesses 

The number of students involved in the project’s two cycles was low. Only nine students of a total of 53 students were also active on the selected ward. Nevertheless, the effects of these students on patient care are very clear. As this was an elective for medical students, voluntary participation presupposes a pre-existing interest in interprofessional cooperation. This may have influenced the evaluation following the teaching and learning unit [51]. However, this does not apply to the nursing students, as their participation in the teaching unit was compulsory. The discrepancy of the participants in the comparison between pre- and post-evaluation (SoSe 2017 (30 students in total): pre: *n*=28, post: *n*=18) is most likely due to the voluntary nature of the evaluation. 

MNA and GMS were used in this study in selecting the scores for screening the nutritional status of patients in the pre- and post-interventional survey. In addition to MNA, the guidelines of the ESPEN especially recommend NRS 2002 for use on wards. However, NRS 2002 seemed to be too imprecise to us. Therefore, in addition to MNA, GMS, which was only published in 2016 [39], was used. MNA was designed for patients aged 65 or above [43]. This also allowed us to examine younger patients and to diagnose malnutrition.

One of the project participants was also a member of the clinic in question and was involved in the interprofessional discussion of the screening to be introduced and the pie charts. In this respect, an influence on the results (Rosenthal effect) is possible. However, there were many other decision makers involved who were not part of the conflict of interest between the clinic and the teaching project. It is also conceivable that the institutional consequences might not necessarily have come from the student feedback but from the study itself (Hawthorne effect). However, without the initiation and implementation of the teaching project at the time, there would probably have been no sensitization to malnutrition. The impetus to carry out screening and the use of pie charts were first put in place by the students.

## 5. Conclusion

Implementation of the teaching unit led to the derivation and implementation of measures that help to increase patient safety. The student feedback sensitized decision-makers to the issue of malnutrition. In the future, the use of pie charts as proposed by the students will allow estimation of the actual amount of a patient’s food intake in a meal during their stay on the ward. By routinely screening the nutritional status, nutritional management is awarded new emphasis in patient care. 

## Funding

The project described has been funded by the Robert Bosch Foundation as part of the “Operation Team” funding line on interprofessional learning in health professions since 2016 (approval number: 32.5.A381.0027.0). 

## Ethics Committee vote

There is a positive vote for the present investigation by the Ethics Committee of the Medical Faculty of the Heinrich Heine University Düsseldorf.

## Competing interests

The authors declare that they have no competing interests.

## Supplementary Material

Guide to file and trend analysis

Demographic data of the patient cohorts (pre-/post-interventional)

Modified from: Rüfenacht U, Rühlin M, Imoberdorf R, Ballmer PE. Das Tellerdiagramm: Ein sinnvolles Erfassungsinstrument für ungenügende Nahrungszufuhr bei Patienten im Krankenhaus. Aktuel Ernaehr Med. 2006;31:66-72. [45]

## Figures and Tables

**Table 1:**
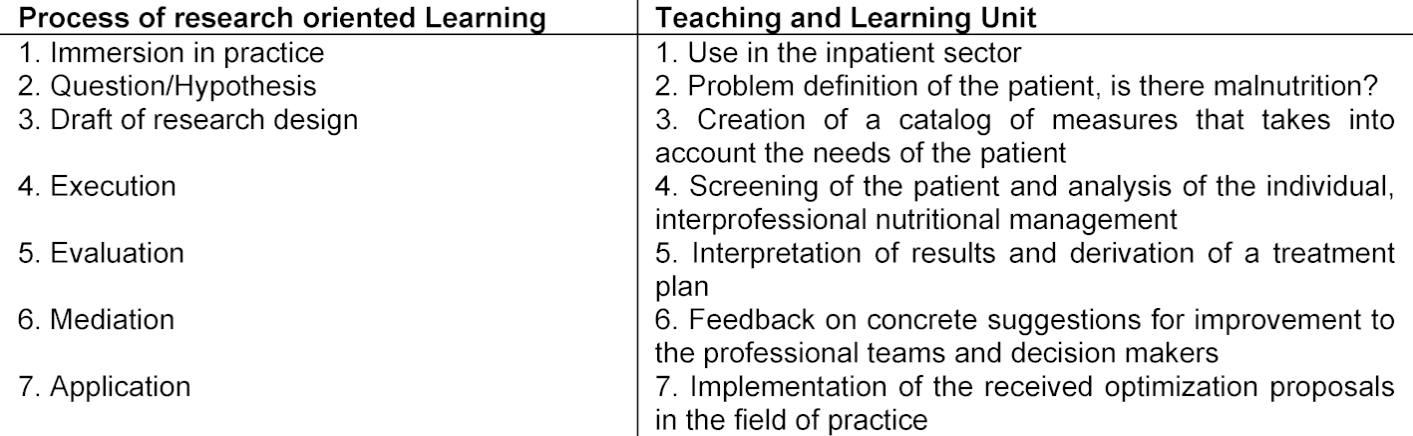
Progression of the teaching and learning unit with reference to the process of research oriented learning according to Wildt [35].

**Table 2:**
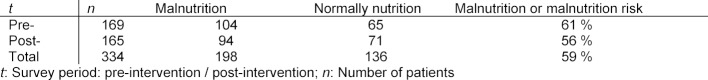
Prevalence of malnutrition in both survey periods

**Table 3:**
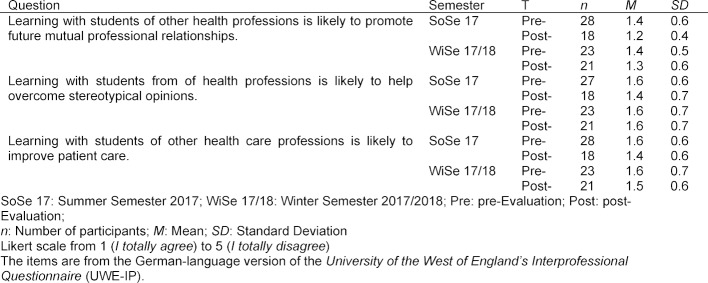
Evaluation results of the participating students (selection with items relevant for the examination)

## References

[1] Leonard M, Graham S, Bonacum D (2004). The human factor: The critical importance of effective teamwork and communication in providing safe care. Qual Saf Health Care.

[2] Vyt A (2008). Interprofessional and transdisciplinary teamwork in health care. Diabetes Metab Res Rev.

[3] Manojlovich M, DeCicco B (2007). Healthy work environments, nurse-physician communication, and patients' outcomes. Am J Crit Care.

[4] Walkenhorst U, Mahler C, Aistleithner R, Hahn EG, Kaap-Fröhlich S, Karstens S, Reiber K, Stock-Schröer B, Sottas B (2015). Position statement GMA Comittee – "Interprofessional Education for the Health Care Professions". GMS Z Med Ausbild.

[5] Martin JS, Ummenhofer W, Manser T, Spirig R (2010). Interprofessional collaboration among nurses and physicians: Making a difference in patient outcome. Swiss Med Wkly.

[6] Miller MA, Kinsel K (1998). Patient-focused care and its implications for nutrition practice. J Am Diet Assoc.

[7] Schmitt MH (2001). Collaboration improves the quality of care: Methodological challenges and evidence from US health care research. J Interprof Care.

[8] Mahdizadeh M, Heydari A, Karimi Moonaghi H (2015). Clinical Interdisciplinary Collaboration Models and Frameworks: From Similarities to Differences: A Systematic Review. Glob J Health Sci.

[9] DeLegge MH, Kelly AT, Kelley AT (2013). State of nutrition support teams. Nutr Clin Pract.

[10] Solomon P (2010). Inter-professional Collaboration: Passing Fad or Way of the Future?. Physiother Can.

[11] Herath C, Zhou Y, Gan Y, Nakandawire N, Gong Y, Lu Z (2017). A comparative study of interprofessional education in global health care: A systematic review. Medicine (Baltimore).

[12] Zwarenstein M, Goldman J, Reeves S (2009). Interprofessional collaboration: effects of practice-based interventions on professional practice and healthcare outcomes. Cochrane Database Syst Rev.

[13] San Martín-Rodríguez L, Beaulieu MD, D'Amour D, Ferrada-Videla M (2005). The determinants of successful collaboration: a review of theoretical and empirical studies. J Interprof Care.

[14] Oandasan I, Reeves S (2005). Key elements for interprofessional education. Part 1: the learner, the educator and the learning context. J Interprof Care.

[15] Mahler C, Karstens S, Roos M, Szecsenyi J (2012). Interprofessionelle Ausbildung für eine patientenzentrierte Versorgung der Zukunft. Die Entwicklung eines Kompetenzprofils für den Bachelor-Studiengang "Interprofessionelle Gesundheitsversorgung". Z Evid Fortbild Qual Gesundhwes.

[16] Foth T, Block K, Stamer M, Schmacke N (2015). The Long Way Toward Cooperation: Nurses and Family Physicians in Northern Germany. Glob Qual Nurs Res.

[17] WHO (2010). Health Professions Network Nursing and Midwifery Office within the Department of Human Ressouces for Health. Framework for Action on Interprofessional Education & Collaborative Practice.

[18] Oandasan I, Reeves S (2005). Key elements of interprofessional education. Part 2: factors, processes and outcomes. J Interprof Care.

[19] Marletta G, Artioli G, Sarli L, Mancini T (2015). The hypothesis of contact in nursing: A narrative review of the literature. Acta Biomed.

[20] Hean S, Dickinson C (2005). The Contact Hypothesis: An exploration of its further potential in interprofessional education. J Interprof Care.

[21] Allport G (1954). The Nature of Prejudice.

[22] Hammick M, Freeth D, Koppel I, Reeves S, Barr H (2007). A best evidence systematic review of interprofessional education: BEME Guide no. 9. Med Teach.

[23] Cichon I, Klapper B (2017). Interprofessionelle Ausbildungsansätze in der Medizin. Bundesgesundheitsbl Gesundheitsforsch Gesundheitsschutz.

[24] Lapkin S, Levett-Jones T, Gilligan C (2013). A systematic review of the effectiveness of interprofessional education in health professional programs. Nurse Educ Today.

[25] Bauer JM, Volkert D, Wirth R, Vellas B, Thomas D, Kondrup J, Pirlich M, Werner H, Sieber CC (2006). Diagnostik der Mangelernährung des älteren Menschen: Ergebnisse eines internationalen Experten-Meetings der BANSS-Stiftung. Dtsch Med Wochenschr.

[26] White JV, Guenter P, Jensen G, Malone A, Schofield M (2012). Consensus statement of the Academy of Nutrition and Dietetics/American Society for Parenteral and Enteral Nutrition: characteristics recommended for the identification and documentation of adult malnutrition (undernutrition). J Acad Nutr Diet.

[27] Kondrup J, Allison SP, Elia M, Vellas B, Plauth M (2003). ESPEN guidelines for nutrition screening 2002. Clin Nutr.

[28] Ordo-ez AM, Madalozzo Schieferdecker ME, Cestonaro T, Cardoso Neto J, Ligocki Campos AC (2013). Nutritional status influences the length of stay and clinical outcomes in patients hospitalized in internal medicine wards. Nutr Hosp.

[29] Venzin RM, Kamber N, Keller WCF, Suter PM, Reinhart WH (2009). How important is malnutrition? A prospective study in internal medicine. Eur J Clin Nutr.

[30] Cederholm T, Barazzoni R, Austin P, Ballmer P, Biolo G, Bischoff SC, Compher C, Correia I, Higashiguchi T, Holst M, Jensen GL, Malone A, Muscaritoli M, Nyulasi I, Pirlich M, Rothenberg E, Schindler K, Schneider SM, de van der Schueren MA, Sieber C, Valentini L, Yu JC, Van Gossum A, Singer P (2017). ESPEN guidelines on definitions and terminology of clinical nutrition. Clin Nutr.

[31] Volkert D, Chourdakis M, Faxen-Irving G, Frühwald T, Landi F, Suominen MH, Vandewoude M, Wirth R, Schneider SM (2015). ESPEN guidelines on nutrition in dementia. Clin Nutr.

[32] Jensen GL, Mirtallo J, Compher C, Dhaliwal R, Forbes A, Grijalba RF, Hardy G, Kondrup J, Labadarios D, Nyulasi I, Castillo Pineda JC, Waitzberg D, International Consensus Guideline Committee (2010). Adult starvation and disease-related malnutrition: a proposal for etiology-based diagnosis in the clinical practice setting from the International Consensus Guideline Committee. JPEN J Parenter Enteral Nutr.

[33] Valente da Silva HG, Santos SO, Silva NO, Ribeiro FD, Josua LL, Moreira AS (2012). Nutritional assessment associated with length of inpatients' hospital stay. Nutr Hosp.

[34] Dang TM, Maggio LA (2017). Supporting the Call to Action: A Review of Nutrition Educational Interventions in the Health Professions Literature and MedEdPORTAL. Acad Med.

[35] Wildt J (2009). Forschendes Lernen: Perspektiven eines Konzeptes: Lehrangebote, Beratungsangebote, Informationen, Tipps, Themen. J Hochschuldidaktik.

[36] Pollard K UWE-IP University of the West of England Interprofessional Questionnaire. Übersetzung durch die Abteilung Allgemeinmedizin und Versorgungsforschung des Universitätsklinikums Heidelberg.

[37] Nestlé Nutrition Institute (2017). Nestlé Nutrition Institute - MNA® Elderly - Overview.

[38] Arbeitsgemeinschaft Klinische Ernährung Ernährungsscores - Screeningbögen.

[39] Roller RE, Eglseer D, Eisenberger A, Wirnsberger GH (2016). The Graz Malnutrition Screening (GMS): a new hospital screening tool for malnutrition. Br J Nutr.

[40] Kwiecien R, Kopp-Schneider A, Blettner M (2011). Concordance analysis: Part 16 of a series on evaluation of scientific publications. Dtsch Arztebl Int.

[41] Altman DG (1991). Practical statistics for medical research.

[42] Volkert D (2017). Ernährung bei Demenzerkrankungen. Internist (Berl).

[43] Vellas B, Guigoz Y, Garry PJ, Nourhashemi F, Bennahum D, Lauque S, Albarede JL (1999). The Mini Nutritional Assessment (MNA) and its use in grading the nutritional state of elderly patients. Nutrition.

[44] Bounoure L, Gomes F, Stanga Z, Keller U, Meier R, Ballmer P, Fehr R, Mueller B, Genton L, Bertrand PC, Norman K, Henzen C, Laviano A, Bischoff S, Schneider SM, Kondrup J, Schuetz P, Members of the Working Group (2016). Detection and treatment of medical inpatients with or at-risk of malnutrition: Suggested procedures based on validated guidelines. Nutrition.

[45] Rüfenacht U, Rühlin M, Imoberdorf R, Ballmer PE (2006). Das Tellerdiagramm: Ein sinnvolles Erfassungsinstrument für ungenügende Nahrungszufuhr bei Patienten im Krankenhaus. Aktuel Ernahrungsmed.

[46] Milligan FJ (2007). Establishing a culture for patient safety - the role of education. Nurse Educ Today.

[47] Wong BM, Etchells EE, Kuper A, Levinson W, Shojania KG (2010). Teaching quality improvement and patient safety to trainees: A systematic review. Acad Med.

[48] García de Lorenzo A, Álvarez Hernández J, Planas M, Burgos R, Araujo K (2011). Multidisciplinary consensus on the approach to hospital malnutrition in Spain. Nutrición Hospitalaria.

[49] Cederholm T, Bosaeus I, Barazzoni R, Bauer J, van Gossum A, Klek S, Muscaritoli M, Nyulasi I, Ockenga J, Schneider SM, de van der Schueren MA, Singer P (2015). Diagnostic criteria for malnutrition - An ESPEN Consensus Statement. Clin Nutr.

[50] Mamhidir AG, Karlsson I, Norberg A, Mona K (2007). Weight increase in patients with dementia, and alteration in meal routines and meal environment after integrity promoting care. J Clin Nurs.

[51] Wershofen B, Heitzmann N, Beltermann E, Fischer MR (2016). Fostering interprofessional communication through case discussions and simulated ward rounds in nursing and medical education: A pilot project. GMS J Med Educ.

