# Supplementary material for: Impact of interprofessional education for medical and nursing students on the nutritional management of in-patients
Source: GMS J Med Educ. 2019 Mar 15;36(2):Doc11. doi: 10.3205/zma001219 (PMC6446465; doi:10.3205/zma001219)
Supplement: Guide to file and trend analysis [file JME-36-2-11-s-001.pdf]

Initials	Gender	DoB	Admission diagnosis	ICD 10	Preliminary diagnosis	ICD 10

GMS	MNA Scr.	MNA Ass.	MNA total

Date of admission	
Date of analysis	

Overall Patient Details:

	Yes(1)/No(0)	if yes	P(1)/Ä(2)/b(3)
Height			
Weight			
BMI			
Living conditions: 1) not alone; 2) living alone; 3) at home but requiring care; 4) in care facility			

Information on patient admission phase days 1-3 on ward ME4:

Assessment with evidence-based tool for malnutrition/undernourishment			
Assessment of nutritional status in the admission phase (days 1-3)			
Medical instructions for further nutritional management			
1) body temperature taken; 2) measured temp. >38°; 3) antibiotic therapy			
1) diarrhea; 2) vomiting; 3) nausea			
Details of hydration state			
Assessment of perceived thirst			
Appetite assessment			

Information on nutrition management on the ward days 1-7:

Food/diet form			
Target calorie intake per day			
Request/consultation on nutritional advice/dietary assistant			
Enteral supplement by mouth			
Enteral nutrition via: 1) Gastric tube; 2) PEG/PEJ			
Parenteral nutrition			
If yes: Documented calorie intake/day			
Documented fasting phases (e.g. prior to surgery, examinations, etc.)			
Eating behavior: 1) independent; 2) bite-sized preparation; 3) in need of full assistance			
Assistance with existing limitation at 1)/2)/3) main meals			
Physical disabilities (plegia, paresis, amputation, etc.), which limit independent food intake			
Mental illnesses (clinically manifest depression, dementia, etc.), which limit independent food intake			
Diseases that require a specific nutritional form<br>(Glycogenoses, lactose intolerance, food allergies, etc.)			
Swallowing disorder			
Chewing disorder			

Diabetes:

1) Type 1; 2) Type 2; 3) other form			
Insulin treatment			
If yes: 1) conventional; 2) intensive treatment			
1) insulin injections by patient; 2) by care staff			
Oral antidiabetic treatment			

Lab results:

Albumin			
Total protein			
Urea			
Creatinin			
Triglycerides			
Total cholesterol			
LDL cholesterol			
HDL cholesterol			

Number of comments and instructions during the ward stay days 1-7:

	Overall numbers
Number of instructions on nutrition management in the medical documentation	
Number of comments on nutritional management in the medical documentation	
Number of comments on nutrition management in nursing documentation	
Number of comments on diet management/counseling through diet/diabetes counseling	

Sample quotes from the documentation on nutrition management:

