# Supplementary material for: Impact of interprofessional education for medical and nursing students on the nutritional management of in-patients
Source: GMS J Med Educ. 2019 Mar 15;36(2):Doc11. doi: 10.3205/zma001219 (PMC6446465; doi:10.3205/zma001219)
Supplement: Demographic data of the patient cohorts (pre-/post-interventional) [file JME-36-2-11-s-002.pdf]

t	*N*	NS	Female<br>*n*	Male<br>*n*	Age<br>*M* (*SD*)
Pre-	169	Total	96	73	68.2<br>(18,8)
Pre-	104	Malnutrition	64	40	75.0<br>(14.8)
Pre-	65	normal nutrition	32	33	57.2<br>(19.4)
Post-	165	Total	93	72	64.6<br>(20.5)
Post-	94	Malnutrition	55	39	71.6<br>(17.4)
Post-	71	normal nutrition	38	33	55.2<br>(20.7)
Pre- + Post-	334	Total	189	145	66.4<br>(19.7)

t: Survey period: pre-intervention/post-intervention; n: Number of patients; NS: Nutritional status; M: Mean; SD: Standard Deviation
