# Supplementary figures and images for: Impact of interprofessional education for medical and nursing students on the nutritional management of in-patients
Source: GMS J Med Educ. 2019 Mar 15;36(2):Doc11. doi: 10.3205/zma001219 (PMC6446465; doi:10.3205/zma001219)

Feeding protocol for:

Date: \_\_\_\_\_

Morning

Midday

Evening

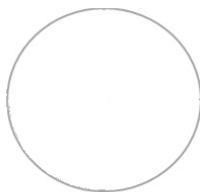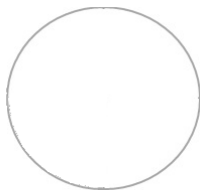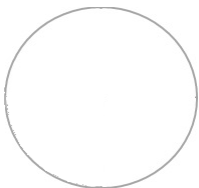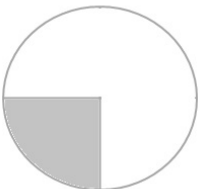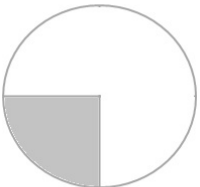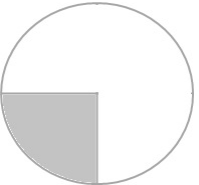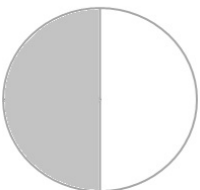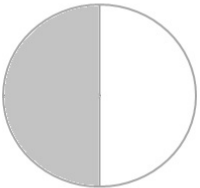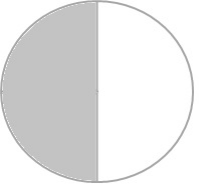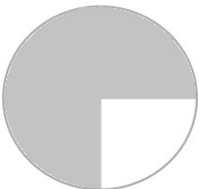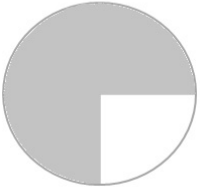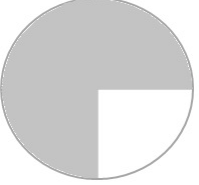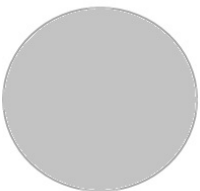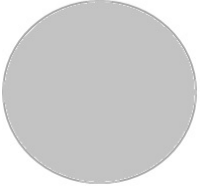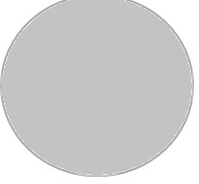

Frequency

Frequency

Frequency

Supplement: Modified from: Rüfenacht U, Rühlin M, Imoberdorf R, Ballmer PE. Das Tellerdiagramm: Ein sinnvolles Erfassungsinstrument für ungenügende Nahrungszufuhr bei Patienten im Krankenhaus. Aktuel Ernaehr Med. 2006;31:66-72. [45] [file JME-36-2-11-s-003.pdf]
